# An atypical role for the myeloid receptor Mincle in central nervous system injury

**DOI:** 10.1177/0271678X16661201

**Published:** 2016-01-01

**Authors:** Thiruma V Arumugam, Silvia Manzanero, Milena Furtado, Patrick J Biggins, Yu-Hsuan Hsieh, Mathias Gelderblom, Kelli PA MacDonald, Ekaterina Salimova, Yu-I Li, Othmar Korn, Deborah Dewar, I Mhairi Macrae, Robert B Ashman, Sung-Chun Tang, Nadia A Rosenthal, Marc J Ruitenberg, Tim Magnus, Christine A Wells

**Affiliations:** 1 Department of Physiology, Yong Loo Lin School of Medicine, National University of Singapore, Singapore; 2 School of Biomedical Sciences, The University of Queensland, Brisbane, Australia; 3 Australian Institute for Bioengineering and Nanotechnology, The University of Queensland, Brisbane, Australia; 4 Australian Regenerative Medicine Institute, Monash University, Melbourne, Australia; 5 The Jackson Laboratory, Bar Harbor, ME, USA; 6 Department of Neurology, University Hospital Hamburg-Eppendorf, Hamburg, Germany; 7 Queensland Institute for Medical Research, Herston, Brisbane, Australia; 8 Department of Pathology and Department of Neurology, National Taiwan University Hospital and National Taiwan University College of Medicine, Taipei, Taiwan; 9 Institute of Neuroscience & Psychology, Wellcome Surgical Institute, University of Glasgow, Glasgow, UK; 10 School of Dentistry, The University of Queensland, Brisbane, Australia; 11 Department of Neurology, Stroke Center, National Taiwan University Hospital, Taipei, Taiwan; 12 Queensland Brain Institute, The University of Queensland, Brisbane, Australia; 13 Faculty of Medicine, Department of Anatomy and Neuroscience, The University of Melbourne, Australia

**Keywords:** C-type lectin, ischemia, middle cerebral artery occlusion, microglia, sterile inflammation

## Abstract

The C-type lectin Mincle is implicated in innate immune responses to sterile inflammation, but its contribution to associated pathologies is not well understood. Herein, we show that Mincle exacerbates neuronal loss following ischemic but not traumatic spinal cord injury. Loss of Mincle was beneficial in a model of transient middle cerebral artery occlusion but did not alter outcomes following heart or gut ischemia. High functional scores in Mincle KO animals using the focal cerebral ischemia model were accompanied by reduced lesion size, fewer infiltrating leukocytes and less neutrophil-derived cytokine production than isogenic controls. Bone marrow chimera experiments revealed that the presence of Mincle in the central nervous system, rather than recruited immune cells, was the critical regulator of a poor outcome following transient middle cerebral artery occlusion. There was no evidence for a direct role for Mincle in microglia or neural activation, but expression in a subset of macrophages resident in the perivascular niche provided new clues on Mincle's role in ischemic stroke.

## Introduction

Ischemic stroke results in the loss of neurons in the perfusion territory of the affected blood vessel via neurodegenerative mechanisms and concomitant infiltration of activated leukocytes.^1^ Chronic inflammatory conditions or infection increase the risk of stroke, and worsen outcome.^2,3^ Molecular models of innate immune signaling show that inflammatory modulation can confound outcomes in stroke. For example, blocking the toll-like receptor (TLR), immune receptor family limits tissue damage.^4,5^ However, targeting immune signaling via MyD88 provided no benefit for neurological outcomes, but worsened neuronal cell death in animals subjected to global or focal ischemia.^6,7^ In mouse models of spinal cord injury (SCI), blocking Tlr2 or Tlr4 reduced microglia activation^8^ but worsened tissue damage and recovery.^9^ The presence of TLRs in the central nervous system (CNS) is suggested to be neuroprotective, whereas increased expression of TLR2 and 4 in peripheral leukocytes is concordant with higher inflammatory markers in clinical stroke and is predictive of poor outcome in stroke patients.^10^

High circulating neutrophil numbers are positive predictors of stroke severity.^11^ Strategies that reduce neutrophil influx to the site of ischemic injury reduce inflammation, collateral blood vessel occlusion and the severity of injury.^12–14^ However, blocking the phagocytic clearance of dead cells by microglia will exacerbate injury,^15^ and reparative roles of tissue macrophages are necessary for wound healing and functional recovery from sterile inflammation.^16^ This suggests that functional differences in infiltrating or resident myeloid cells during the acute phase of sterile injury are important determinants of recovery from stroke.

Mincle (*Clec4e*) is a myeloid receptor that has been reported to recognize necrotic cells via detection of the nuclear protein Sap130.^17^ Mincle regulates inflammatory responses to fungal and mycobacterial pathogens^18–21^ via spleen tyrosine kinase (Syk), and the caspase recruitment domain protein Card9.^22,23^ Inhibition of Syk limits thrombosis and vascular inflammation in a variety of animal models of sterile injury;^24^ therefore, the Mincle/Syk axis is likely to also contribute to the pathophysiology of ischemic stroke.

Two previous studies used Syk inhibition to suggest a deleterious role for Mincle in subarachnoid haemorrhage^25^ and ischemic stroke,^26^ but as Syk is not selective, the role of Mincle in stroke remains poorly defined. In the current study, we used the *Clec4e*^*−/−*^ mouse, together with bone marrow chimeras, to demonstrate an atypical role for Mincle in ischemic stroke outcomes. Using the KO mouse to determine antibody specificity, we demonstrate that Mincle does not have the widespread brain expression previously described,^25,26^ but instead is restricted to perivascular macrophages and peripheral leukocytes. Absence of Mincle did not affect outcomes following traumatic SCI, or of ischemic injuries in other organs, such as the heart or the intestine. The combined data presented here suggest a key role for Mincle in ischemic CNS injuries, where the integrity of the blood–brain/spinal cord barrier is not compromised by mechanical forces during the initiating event.

## Materials and methods

### Animals

All experimental procedures followed the “Australian code of practice for the care and use of animals for scientific purposes,” approved by The University of Queensland and Monash University Animal Ethics Committees (ethics license numbers SBMS/358/12/NHMRC/ARC, SBMS/085/09, MARP-2011-175 and SBMS/311/12/SPINALCURE), and further husbandry details, including ARRIVE guidelines for reporting animal research are available in the supplementary methods.

Homozygous null C57Bl/6J *Clec4e*^*−/−*^ mice were used as previously described,^21^ and they were compared to a control cohort of isogenic C57BL/6J or cohoused *Clec4e*^*+/−*^ littermates, which exhibited equivalent immune phenotypes in this and previous studies.^27^ Full details on animal numbers assigned to each experiment are described in the supplementary methods. Unless otherwise stated, a randomized experimental designed was used, where littermates (*Clec4e*^+/−^ or *Clec4e*^−/−^) were cohoused and animals were selected at random by an operator who was blind to the genotype of the animals and the analysis of material was conducted in a double-blinded manner.

### Focal cerebral ischemia model

**Figure 1.**
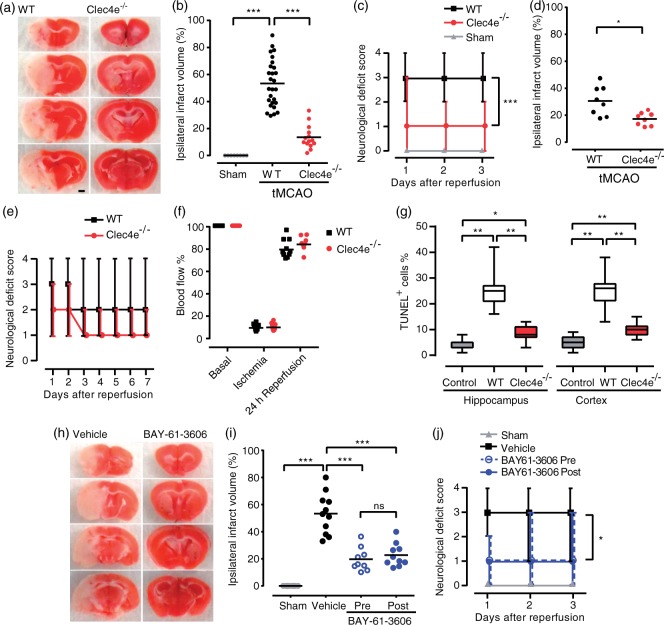
Mincle signaling worsens brain damage and functional outcome after transient MCAO. Mice of the indicated genotypes were subjected to sham surgery or transient middle cerebral artery occlusion (tMCAO), and brain damage and neurological function were evaluated (a) Representative TTC-stained brain sections from WT and *Clec4e*^*−/−*^ mice three days post-reperfusion. Infarct volumes (b) for WT mice subjected to sham surgery (n = 6), and WT (n = 27) and *Clec4e*^*−/−*^ (n = 14) mice three days after tMCAO. Bars represent mean. (c) The corresponding daily neurological deficit scores are shown as median and range. (d) Infarct volumes for WT (n = 8) and *Clec4e*^*−/−*^ (n = 8) mice seven days after tMCAO are significantly different as shown by t-test. (e) The corresponding daily neurological deficit scores are shown as grouped with median and range. (f) Laser Doppler flowmetry shows no differences between samples in the extent to which MCAO compromises blood flow. (g) TUNEL-positive cells were quantified in both hippocampus and cortex from control (n = 4), WT (n = 6) and *Clec4e*^*−/−*^ (n = 6) mice after global cerebral ischemia. The percentage of TUNEL-positive cells is represented showing median, the 25th to 75th percentiles, and min-max range. (h) Representative TTC-stained brain sections from mice treated with vehicle or the Syk inhibitor BAY-61-3606, three days post-reperfusion. (i) Infarct volumes for WT mice subjected to sham surgery (n = 6), vehicle-treated (n = 11) mice, or mice treated with BAY-61-3606 before MCAO (n = 9) or 3 h after the onset of reperfusion (n = 10), three days after tMCAO. (j) The corresponding daily neurological deficit scores are as median and range, and both pre- and post-treated samples are significantly different from vehicle control. Tests: (b, i, g) ANOVA, (c, j) Kruskal–Wallis, (d) t-test, (e) Mann–Whitney. ***: *p* < 0.001, **: *p* < 0.01, *: *p* < 0.05, ns: not significant; comparisons indicated by brackets. Scale bar for images: 1 mm.

**Figure 2.**
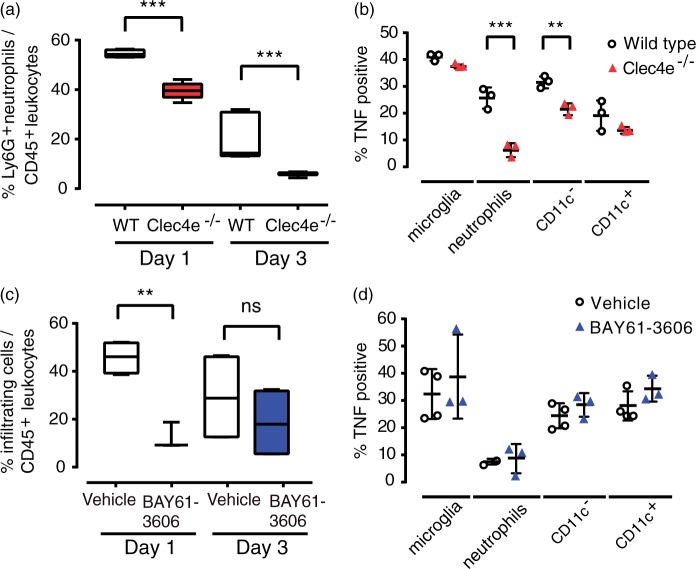
Neutrophil infiltration was significantly reduced in *Clec4e*^*−/−*^ mice following tMCAO. (a) Flow cytometry of leukocytes in the ipsilateral hemisphere showed a significantly lower proportion of infiltrating neutrophils (CD45^high^, CD11b^+^, Ly6G^+^) in *Clec4e*^*−/−*^ mice at one and three days post-reperfusion. (b) There were fewer TNF-positive infiltrating neutrophils and CD11c^*−*^ monocytes in *Clec4e*^*−/−*^ compared to WT mice 24 h after reperfusion. (c) Flow cytometry revealed a significantly lower proportion of infiltrating leukocytes (CD45^high^, CD11b^+^) in *Clec4e*^*−/−*^ mice than in WT controls 24 h post-reperfusion. (d) No differences in the proportion of infiltrating neutrophils or in TNF-positive leukocytes were observed. t-test: ***: *p* < 0.001, **: *p* < 0.01, ns: not significant. Comparisons indicated by brackets. % cells in (a) and (c) are represented by box and whiskers plots, showing median, the 25th to 75th percentiles, and min-max range. % TNF-positive cells are represented by mean + s.e.m.

**Figure 3.**
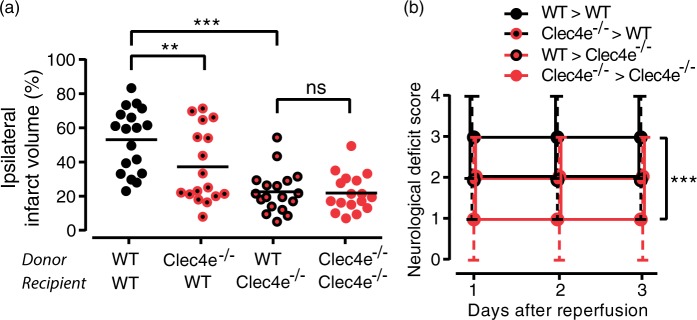
The origin of the *Clec4e*^*−/−*^ protective effect is in the central nervous system. (a) Ipsilateral infarct volume three days after tMCAO from bone marrow chimeras obtained between WT (C57BL/6J mice carrying the CD45.1 allele) and *Clec4e*^*−/−*^ animals. Donor and recipient genotypes indicated on the X-axis. Bars represent median and range. (b) Neurological deficit scores of bone marrow chimeras, assessed daily for three days, shown as median and range, where both Clec4e^*−*/*−*^ recipient samples are significantly different from WT>WT sample. WT donor to WT recipient, n = 18; *Clec4e*^*−/−*^ donor to WT recipient, n = 17; WT donor to *Clec4e*^*−/−*^ recipient, n = 18; *Clec4e*^*−/−*^ donor to *Clec4e*^*−/−*^ recipient, n = 17. ANOVA: ***: *p* < 0.001, **: *p* < 0.01, ns: not significant. Comparisons indicated by brackets.

**Figure 5.**
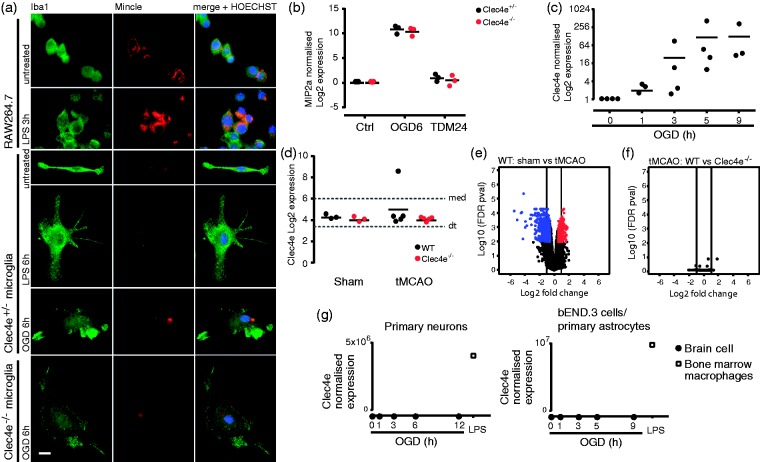
Mincle could not be found in microglia, neurons, astrocytes or endothelial cells. (a) An antibody that detects Mincle in the mouse macrophage cell line RAW264.7, and shows its upregulation by LPS, does not detect Mincle in cultured microglia in basal conditions or stimulated with LPS or oxygen and glucose deprivation (OGD). Scale bar: 10 µm. (b) MIP2a qRT-PCR in *Clec4e*^*+/−*^ and *Clec4e*^*−/−*^ cultured microglia from neonatal mice shows that Mincle does not contribute to *MIP2a* gene expression in microglia, and Mincle sufficient microglia do not produce *MIP2a* in response to the Mincle-specific ligand trehalose dimycolate (TDM). Data points represent different cultures, each derived from a single mouse (c) Ex vivo microglia upregulate *Clec4e* expression under OGD, as measured by qRT-PCR. (d) WT microglia sorted from the ipsilesional brain hemisphere 24 h after tMCAO do not show *Clec4e* upregulation, as shown by microarray, sham n = 3 per genotype, tMCAO n = 5 per genotype (dt: detection threshold, 3.38; med: median, 6) (e, f) Volcano plots for differences in microglial gene expression between sham surgery and tMCAO for WT mice (e), and between WT and *Clec4e*^*−/−*^ tMCAO (f), show that, whether there are notable differences in microglial gene expression between sham surgery and tMCAO followed by 24 h reperfusion, *Clec4e* is not a contributing factor. Red dots: > 1.5 upregulated in the first sample mentioned above graph (*p* < 0.05); blue dots: > 1.5 downregulated in the first sample mentioned above graph (*p* < 0.05). (g) Absence of *Clec4e* mRNA in primary cortical neurons (left), cells of the brain endothelial cells line bEND.3 and primary astrocytes (right), untreated or under a time course of OGD (n = 3). RNA from WT bone marrow-derived macrophages treated with LPS for 3 h is the positive control for all samples (n = 1).

Male, three to six-month-old mice were anesthetized for focal cerebral ischemia by transient middle cerebral artery occlusion (tMCAO). The first round of surgery (Figures 1 and 2) did not use a randomized experimental design. For all other tMCAO, including bone marrow chimeras (Figure 3, n = 70), and microglia profiling (Figure 5, n = 16), operators were blinded to genotype or treatment group and randomization was based on predesigned lists using color-coded cages and reagents. Exclusion criteria were excessive bleeding or death within 24 h after tMCAO. Details of the tMCAO surgery have been published previously^4,5^ and are provided in the supplementary methods. Animals were subjected to cerebral blood flow measurements using a laser Doppler perfusion monitor (Moor Lab) placed perpendicular to the surface of the right parietal skull (1 mm posterior and 5 mm lateral to the bregma) to monitor blood flow in the MCAO territory.

Syk inhibitor BAY61-3606 (B9685, Sigma-Aldrich) was injected (femoral vein), 1 mg/kg (25 μl) 30 min before tMCAO, or 3 h post-reperfusion.

### Quantification of cerebral infarction and neurological deficit assessment

Infarct areas were calculated at three or seven days post-reperfusion from digitized sections stained with 2% 2,3,5-triphenyltetrazolium chloride (TTC, T8877 Sigma-Aldrich) using NIH image 6.1 software. To correct for brain swelling, the infarct area was determined by subtracting the area of undamaged tissue in the left (ipsilateral) hemisphere from that of the whole contralateral hemisphere. Infarct volume was calculated by integration of infarct areas for all slices of each brain, and then expressed as a % of the ipsilateral hemisphere. The functional consequences of tMCAO were evaluated using a 5-point neurological deficit score (0, no deficit; 1, failure to extend right paw; 2, circling to the right; 3, falling to the right; and 4, unable to walk spontaneously^28^).

### Global ischemia model and TUNEL assay

Adult male, age-matched C57BL6/J WT (n = 4) and *Clec4e*^*−/−*^ mice (n = 6) were used. Transient global cerebral ischemia was performed by the two-vessel occlusion model^1^ and described in more detail in the supplementary methods. The In Situ Cell Death Detection Kit, POD (11684817910, Roche, Switzerland) was used according to manufacturer's instructions, and tissue was counterstained with Gills hematoxylin. Sections treated with 100 U/ml recombinant DNase I were used as positive control.

### Flow cytometry

Protocols for fixation and perfusion of brains after tMCAO have been previously described,^29^ and details provided in the supplementary methods. In brief, each experiment consisted of a pool of three lesioned or ipsilateral hemispheres. Cellular fractions were isolated from collagenase digested tissue, separated from myelin and debris, then incubated appropriate antibodies (see supplementary methods). Data were acquired with a LSR II FACS system (BD Biosciences) and analyzed with FlowJo (TreeStar). Doublets were excluded with FSC-A and FSC-H linearity.

### Generation of bone marrow chimeric mice

Recipient mice (three to six months of age) were sub-lethally irradiated with 2 × 5 Gy, 14 h apart. Immune system rescue via bone marrow transplantation from either WT or *Clec4e*^*−/−*^ donors, 3–4 h after the second dose of irradiation. Chimeric mice recovered > 3 months for full reconstitution of the peripheral immune compartment, confirmed as >95% donor engraftment efficiency by relative frequency of donor and host white blood cells, on the basis of CD45.1^+^/ CD45.2^+^ antigen expression. Mice were color coded prior to surgery and subjected to tMCAO by an experimenter blinded to the genotype or treatment groups. Infarct size was determined as detailed above.

### SCI and assessment of locomotor recovery

Adult, age- and weight-matched female C57BL6/J WT (n = 12) and *Clec4e*^*−/−*^ mice (n = 14) were used for these experiments. Order of surgery was randomized based on predesigned lists, with the experimenter conducting the surgery remaining blinded to genotype throughout all aspects of surgery. A severe contusive SCI as previously described is given with full details in the supplementary methods.^30–32^ Briefly, the procedure involved a dorsal laminectomy and a force-controlled 70 kilodyne (kd) impact applied at spinal level T11, using the Infinite Horizon impactor device (precision systems and instrumentation). Recovery of hind-limb function was assessed using the 10-point Basso Mouse Scale (BMS), a system designed specifically for the assessment of murine locomotor recovery following SCI.^33^ Multiple aspects of locomotion, including ankle movement, stepping, co-ordination, paw placement, trunk stability and tail position were assessed using this scale. Experimental animals were randomly picked up from their cages and assessed by two investigators blinded to the genotype at one, four, seven days post-SCI and then weekly thereafter up until the study endpoint (35 days post-injury). Animals deviating > ±5 kdyne from the mean force, or spinal cord tissue displacement ±100 µm from the experimental mean were excluded. For the remaining n = 10 mice per genotype, the actual applied force and displacement for WT and *Clec4e*^*−/−*^ animals were 75.40 ± 0.91 vs. 74.10 ± 0.99 kdyne (*p* > 0.34), and 537.7 ± 15.22 vs. 535.9 ± 18.11 µm, respectively (*p* > 0.94).

### Spinal cord tissue sectioning and immunofluorescence

In brief, mice were euthanized at day 35 post-injury. Transverse 20 μm thick sections of spinal cord were cut, processed and stained with appropriate antibodies as described in the supplementary methods. Images were captured on a single plane using a Zeiss Axio Imager and Zen Blue 2012 Software (Zeiss), and analyzed with ImageJ software. Section areas were determined by outlining the section boundary on the GFAP^+^ channel (excluding the leptomeninges). Proportional area measurements were calculated by thresholding the FluoroMyelin Red stained area in ImageJ and dividing it by the total section area. Lesion volumes and/or length were calculated by multiplying fibronectin^+^ areas by the section thickness and 1:5 series count.

### Intestinal ischemia and reperfusion, histological analysis and myeloperoxidase quantification

Details on surgical procedures are provided in the supplementary methods. Briefly, the surgeries were not randomized, but tissues were collected into coded tubes and the analysis performed by an operator blind to genotype. A ligature was tied with silk suture material around the superior mesenteric artery except in animals undergoing sham surgery. After 30 min of ischemia, the ligature was removed, and after 2 h of reperfusion, the mice were euthanized. For histological analysis, three portions of small intestine were stored in 4% paraformaldehyde for 24 h. Tissues were embedded in paraffin wax, sectioned transversely and stained with hematoxylin/eosin. The average of villi damage was determined after grading each of 100 villi per mouse on a 0–6 scale as previously described.^34^ For MPO activity, three portions of small intestine were homogenized in 50 mM potassium phosphate, centrifuged and pellets resuspended in 0.25 mM hexadecyltrimethylammonium bromide (H5882, Sigma-Aldrich) for MPO solubilization. After homogenization and centrifugation, supernatants were assayed with 1.21 mg/ml o-dianisidine dihydrochloride (D3252, Sigma-Aldrich) and 2.17% hydrogen peroxide, and absorbance read at 460 nm.

### Myocardial infarction and echocardiography analysis

All animals in the same cage (siblings) were experimented on in a blinded fashion (for both echocardiography and surgery). Genotypes were checked after the experiment was finalized. To induce myocardial infarction, the left coronary artery (LDCA) of 10-week-old mice was ligated. For non-invasive echocardiography, in homeostasis or one month after surgical intervention, mice were anaesthetized and kept sedated under 1.5% isoflurane. Imaging was performed in spontaneously breathing animals in prone position using Vevo 2100 Imaging System (FUJIFILM VisualSonics) equipped with 18 to 38 MHz linear array transducer. Standard parasternal long- and short-axis views were obtained to assess left ventricular chamber function. Function was calculated using Vevo2100 Cardiac Measurements Package. Mice were euthanized one month after surgery, and their hearts dissected for histology.

### Cell culture, and oxygen and glucose deprivation

The murine brain endothelial cell line bEnd.3 (CRL-2299) and the murine macrophage line RAW264.7 cells (TIB-71) were obtained from ATCC. Neuronal cultures were established from littermate 16-day-old WT, *Clec4e*^*+/−*^ or *Clec4e*^*−/−*^ mouse embryos, and ascertained by immunofluorescence to be 95% neurons and 5% astrocytes. Glial cultures were established from postnatal day 1 WT, Clec4e^+/*−*^ or Clec4e^*−*/*−*^ mice. Microglia were separated from astrocytes with the use of CD11b (Microglia) MicroBeads (Miltenyi Biotec). For oxygen and glucose deprivation (OGD), cultures were incubated with glucose-free Locke's buffer (in mmol/L: 154 NaCl, 5.6 KCl, 2.3 CaCl_2_, 1 MgCl_2_, 3.6 NaHCO_3_, 5 HEPES, pH 7.2, supplemented with gentamycin 10 mg/L) placed in an incubator, where the oxygen was displaced with nitrogen to a level of 0.2%, and incubated for 3 h. Incubation with trehalose 6,6 dimycolate (Sigma Aldrich) was conducted for 24 h.

### Microglia isolation and microarray

WT and *Clec4e*^*−/−*^ mice were coded and randomized for tMCAO surgery and FACs profiling as described above, and in supplementary methods. Microglia were isolated 24 h post tMCAO. For each profiled replicate, two ipsilesional hemispheres (with cerebellum and brainstem removed) were pooled for microglia isolation. For sham-operated animals, the whole forebrain was used and brains were not pooled. After myelin separation by Percoll gradient centrifugation, 80,000 CD45^intermediate^, CD11b^+^ microglial cells were sorted from each sample. Doublets were excluded with FSC-A and FSC-H linearity, and dead cells excluded using Zombie Violet™ Fixable Viability Kit (423113, BioLegend). RNA was isolated as described above. Samples were amplified (GeneChip WT Pico Kit 902623, Affymetrix) and processed with the Mouse 2.0ST Gene Array WT pico assay (902463, Affymetrix) by the Ramaciotti Centre for Genomics, University of New South Wales. The expression data (RMA background corrected, quantile normalized) are hosted by www.stemformatics.org (dataset S4M-6731)^35^ and is available from GEO (Accession GSE77986). The expression threshold was calculated as the median expression of all antigenomic probesets on the microarray (log_2_ 3.38). Probes that failed to be expressed above detection threshold in the majority of biological replicates in at least one comparison group were removed from the analysis. The R/Bioconductor *limma*^36^ package was used to find differentially expressed genes (DEG) at FDR *p* < 0.01.

### Identification of Mincle-expressing cells by IF

Mouse cultured cells were fixed in 4% paraformaldehyde; 6 μm microtome sections from Wistar Kyoto or spontaneously hypertensive stroke-prone (SHRSP) rats subjected to permanent MCAO for 24 h using diathermy with modification were obtained from a previous study.^37^ Antibodies are described in the supplementary methods. Images were acquired using an Olympus BX61 microscope (Japan).

### qRT-PCR

RNA was isolated (RNeasy Plus Mini Kit, 74134, Qiagen), and cDNA synthesized using iScript Reverse Transcription Supermix, Bio-Rad. qPCR was performed with FastStart Universal SYBR Green Master [Rox] (04913850001, Roche). Primer sequences are provided in the supplemental methods.

### Data analysis

Unless indicated otherwise, the experimental unit for in vivo experiments was a single animal. For neurological deficit scores, differences between experimental conditions were assessed using a Kruskal–Wallis test followed by Dunn's multiple comparison test, or Mann–Whitney test. Assessment of variance between all other experimental groups was determined by one-way analysis of variance (ANOVA) followed by Neuman–Keuls multiple comparisons test, or two-tailed Student *t*-test as appropriate, and the specific tests applied are indicated in all figure legends. Unless stated, error bars show standard deviation (s.d.).

## Results

### Mincle deficiency improves functional outcomes and reduces infarct size in mouse models of cerebral ischemia

Mincle knock out mice (*Clec4e*^*−/−*^) were used to assess the impact of Mincle on stroke-induced inflammation and tissue damage following 1 h tMCAO. In all functional and physiological measures, the *Clec4e*^*−/−*^ group had fewer impairments than isogenic WT controls, including 50% smaller infarct volume (TTC staining) at three days post-stroke (Figure 1(a) and (b)), and lower neurological deficit scores (Figure 1(c)). These findings were corroborated in an independent experiment in mice monitored up to seven days post-stroke, with the *Clec4e^−/−^* group recording an overall and significant decrease in infarct size and neurological deficit scores compared to the WT group (Figure 1(d) and (e)). Arterial blood flow, measured by laser Doppler flowmetry during ischemia, was equivalent between the groups (Figure 1(f)) and both genotypes showed comparable recovery 24 h after reperfusion. *Clec4e*^*−/−*^ mice subjected to global cerebral ischemia had significantly fewer TUNEL-positive cells than the control group, indicating loss of Mincle was associated with less apoptosis in hippocampus and cortex (Figure 1(g)).

Mice treated with the Syk inhibitor BAY61-3606 before tMCAO, or 3 h after reperfusion, showed >50% decrease in infarct volume (Figure 1(h) and (i)) and greater functional scores than vehicle-treated mice (Figure 1(j)), supporting the efficacy of Syk inhibitors in this stroke model.

### Mincle and its downstream partner Syk mediate reperfusion-induced leukocyte brain infiltration

Immune cell populations present in ipsilateral brain hemispheres from WT and *Clec4e*^*−/−*^ mice were analyzed by flow cytometry over the first three days post-reperfusion. The recruitment of neutrophils (CD45^high^, CD11b^+^, Ly6G^+^) to the injured brain parenchyma at one or three days after tMCAO was significantly less in *Clec4e*^*−/−*^ than WT mice (Figure 2(a)). Fewer TNF-positive neutrophils, as well as fewer CD11c^*−*^ monocytes (CD45^high^, CD11b^+^, CD11c^*−*^) were observed one day after reperfusion in *Clec4e*^*−/−*^ than control groups (Figure 2(b)). It is noteworthy that microglia (CD45^intermediate^, CD11b^+^), which appeared to be the primary source of TNF in the injured hemisphere, showed no difference in activation between genotypes (Figure 2(b)). A single dose of the Syk inhibitor 3 h after tMCAO also impacted on inflammatory cell recruitment with significantly fewer CD45^high^ cells in the Syk-treated animals compared to the vehicle-treated group on day 1 (Figure 2(c)); however, no differences were recorded between vehicle and inhibitor-treated animals on day 3 after tMCAO. In contrast to the lower numbers of TNF + leukocytes recorded in *Clec4e*^*−/−*^ mice (Figure 2(a) and (b)), no differences in TNF-positive myeloid cells were observed between WT animals treated with Syk inhibitor or vehicle control (Figure 2(d)).

### Ischemia-induced tissue damage is driven by Mincle expression in the brain, not peripheral blood cells

We next aimed to determine whether Mincle influences stroke outcomes via its expression in circulating myeloid cells, CNS-resident cells, or a combination thereof. Bone marrow chimera experiments showed that a lack of Mincle in the peripheral immune compartment (*Clec4e^−/−^ marrow to WT recipient*) had a small but significant effect on infarct volume after tMCAO (Figure 3(a) and (b)), indicating a partial contribution of bone marrow-derived cells to the *Clec4e*^*−/−*^ phenotype. *Clec4e*^*−/−*^ mice that received a WT bone marrow transplant (*WT to Clec4e*^*−/−*^), to reinstate Mincle expression on circulating myeloid but not CNS-resident cells, were equally well protected against tMCAO as the chimeric group with global *Clec4e* deficiency (*Clec4e*^*−/−*^ to *Clec4e*^*−/*^) based on both infarct volumes and neurological deficit scores (Figure 3(a) and (b)). Loss of Mincle on CNS-resident cells thus appears to be the critical contributor towards the protective phenotype observed in *Clec4e*^*−/−*^ mice.

### Mincle deficiency does not influence the outcome from traumatic CNS injury or peripheral organ ischemia

**Figure 4.**
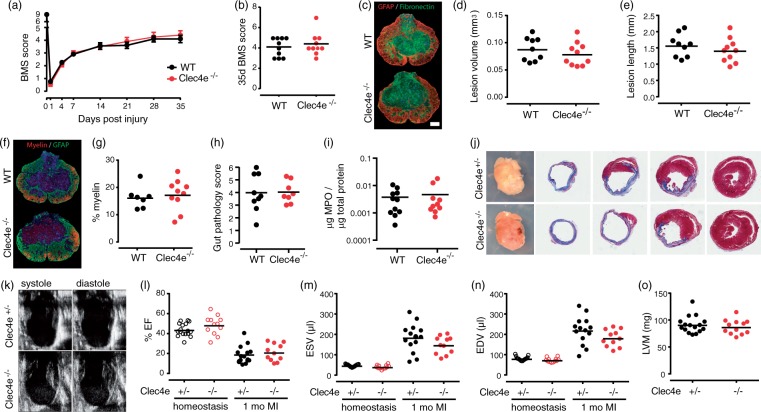
Absence of Mincle does not affect the outcome of spinal cord injury, intestinal ischemia and reperfusion (I/R) or myocardial ischemia. (a) No significant differences between genotypes (n = 10) in 35 days post-injury BMS locomotor scores (ANOVA; *p* > 0.05), or at endpoint (b). (c) Representative GFAP^+^/Fibronectin^+^ staining for the quantification of lesion core in WT and *Clec4e*^*−/−*^ mice. No significant differences were found between genotypes in either lesion core volume (d, *t*-test *p* > 0.05) or length (e, *t*-test *p* > 0.05). Bars represent mean. Scale bar for images: 200 µm. Quantification of myelin staining in WT and *Clec4e*^*−/−*^ spinal cords (f) revealed no significant differences between genotypes (g). Intestinal I/R revealed no differences between genotypes with regards to pathology scores (h) or to levels of myeloperoxidase (MPO) an indicator of neutrophil infiltration (i). Whole mount views and histological sections of control and mutant hearts after one month of infarction (j). Trichrome staining, representative of n = 10, shows scarring (blue) and viable myocardium (red) at various transverse levels of the heart, from apex (left panel) to base (right panel). (k–o) Echocardiography analysis of control and mutant hearts after one month of infarction (*Clec4e*^*+/−*^ and WT animals, n = 14, *Clec4e*^*−/−*^ animals, n = 11). (k) Representative images of infarcted hearts in systole and diastole. Ejection fraction (EF – l), end systolic volume (ESV – m) and end diastolic volume (EDV – n), as well as other analyzed parameters, are not significantly altered in mutant hearts, suggesting that *Clec4e* is not implicated in the regenerative response after myocardial infarction. Left ventricular mass (LVM – o) was used as an indication of heart size in homeostasis and is not significantly different between control and mutant hearts.

We theorized that if Mincle is a general activator in the local response to tissue damage, then it may also play a key role in other models of tissue injury. Lack of Mincle did not result in an altered and/or improved recovery from contusive SCI, with no differences observed between genotypes for hind-limb locomotor performance up to at least 35 days post-injury (Figure 4(a) and (b)), nor did absence of Mincle alter lesion volume, lesion length, or myelin preservation (Figure 4(c) to (g)). We observed no role for Mincle in two models of peripheral ischemic injury; intestinal ischemia/reperfusion, or myocardial infarction. WT and *Clec4e*^*−/−*^ mice subjected to 30 min of ischemia and 2 h of blood reperfusion showed similar gut pathology scores (Figure 4(h)). Neutrophil recruitment to the injured intestine, based on myeloperoxidase (MPO) content in the tissue, was not Mincle-dependent (Figure 4(i)). A similar level of scarring was observed between the hearts of *Clec4e*^*−/−*^ mice (n = 11), *Clec4e*^*+/−*^ littermates and WT animals (combined n = 14) at one month following myocardial infarction (Figure 4(j)). Echocardiography analysis of heart chamber function, either in homeostasis or one month after surgical intervention (Figure 4(k)), revealed no significant differences between genotypes in ejection fraction (Figure 4(l)), or systolic and diastolic end volume (Figure 4(m) and (n)). Left ventricular mass was also similar for both genotypes in homeostasis (Figure 4(o)), confirming a lack of size bias in baseline homeostatic functional parameters.

### Mincle expression in the brain is restricted to a specific cell type

To better understand the unique role of Mincle in ischemic stroke, we performed a careful search for the cell(s) in the CNS expressing Mincle. Given that Mincle is a myeloid cell receptor, we first assessed microglia. Immunofluorescence with 1B6, a rat antibody that recognizes Mincle in the mouse macrophage cell line RAW264.7, showed no expression of Mincle in microglia cultures derived from neonatal brains. Stimulation with OGD as an in vitro model of ischemia also failed to increase Mincle staining (Figure 5(a)). We next determined whether microglia were functionally competent to respond to the Mincle-ligand trehalose dimycolate (TDM), by measuring production of the target chemokine Cxcl2 (*Mip2a*).^19^ Cultured microglia upregulated *Mip2a* expression in response to OGD regardless of their genotype; however, WT (*Clec4e*^*+/−*^) microglia failed to induce *Mip2a* mRNA in response to TDM stimulation, indicating a lack of Mincle-dependant signaling in these cells (Figure 5(b)). *Clec4e*^*+/−*^ primary microglia did upregulate *Clec4e* mRNA in response to OGD (Figure 5(c)), suggesting the possibility that Mincle may play a role in microglia during tMCAO. We therefore sorted microglia (CD45^intermediate^, CD11b^+^) by FACS from the ipsilesional hemispheres of *Clec4e*^*−/−*^ and WT controls, 24 h after either tMCAO or sham surgery, for transcriptome profiling. *Clec4e* mRNA was not higher in WT microglia after tMCAO (Figure 5(d)). As expected, dramatic alterations in gene expression were observed between microglia from tMCAO mice compared to their sham-operated counterparts (Figure 5(e)), but the presence or absence of Mincle itself did not affect microglial gene expression (Figure 5(f)). The observations that Mincle is not expressed by, nor functional in, microglia thus exclude microglia as the primary mediators of the neuroprotective phenotype in *Clec4e*^*−/−*^ mice.

Close evaluation of Mincle expression, using both mRNA and protein analysis on WT and *Clec4e*^*−*/*−*^ mice, also failed to reveal Mincle expression by other cell types such as primary neurons, the brain endothelial cell line bEND.3, or primary astrocytes, including in response to OGD (Figure 5(g)). Mincle mRNA was also not detectable by qPCR in various human neuronal cell lines (CHP-212 or SH-SY5Y), nor under a variety of metabolic deprivation or inflammatory models (data not shown). We were unable to confirm the previously reported cellular pattern of Mincle protein expression in the mouse by immunohistochemistry (IHC), largely because of the poor specificity of anti-Mincle antibodies in mouse: conditions that gave least staining in the *Clec4e*^*−/−*^ sections also lacked staining in WT tissue (Supplementary Figure 1).

### In rat, Mincle is expressed on CD163-positive perivascular macrophages after permanent MCAO

**Figure 6.**
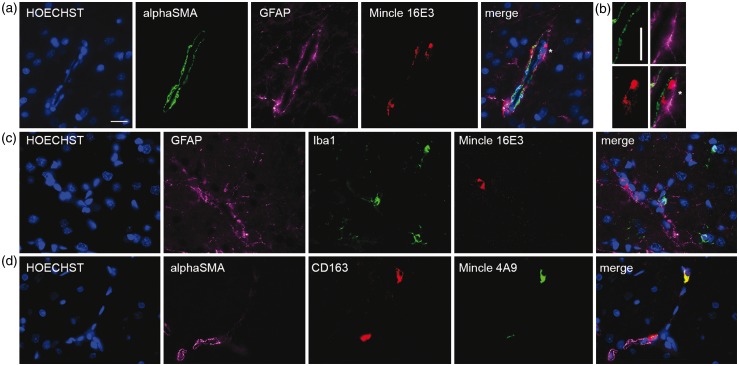
Mincle immunofluorescence in the cortical contralateral (non-infarcted) region of rats subjected to permanent MCAO shows presence of Mincle in the perivascular macrophages. (a) Mouse anti-Mincle antibody 16E3 shows Mincle^+^ cells do not co-localize with pericytes (alpha-SMA^+^) or astrocytes (GFAP^+^), but with other cells located in the periphery of the blood vessels. (b) Detail of the previous cell (* indicates position) showing a Mincle^+^ cell (red) located between pericytes (green) and astrocytes (purple). (c) Mincle^+^ cells do not co-localize with microglia (Iba1^+^). (d) Rat anti-Mincle antibody 4A9 staining shows co-localization with CD163, a marker of perivascular macrophages. Scale bars: 20 µm.

In order to verify our observations that Mincle does not have widespread expression in the brain, we examined rat brain tissue after permanent MCAO.^37^ Throughout the brain, in both infarct and non-ischemic tissue, Mincle^+^ cells were always associated with the vasculature. Monoclonal antibodies 4A9 (rat) and 16E3 (mouse) provided the same result (Figure 6). Mincle was not present in cells positive for the pericyte marker alpha-SMA (Figure 6(a) to (d)), with the astrocyte marker GFAP (Figure 6(a) and (c)) or with the microglial marker Iba1 (Figure 6(c)). Mincle^ + ^cells always appeared sandwiched between pericytes and astrocytes (Figure 6(b)), and were positive for perivascular macrophage marker CD163^37^ (Figure 6(d)). Together these data confirm that Mincle is not directly regulating microglia or astrocyte responses to sterile inflammation.

## Discussion

The resistance of the *Clec4e*^*−/−*^ mice to ischemic brain injury, but not to traumatic SCI, or peripheral (gut or heart) ischemia strongly suggests a unique role for Mincle in the context of cerebral ischemia. Mincle's major role is in the induction, rather than propagation of ischemic inflammation, and the absence of Mincle in the brain prevented the infiltration of peripheral immune cells, reducing the activation of neutrophils and monocytes after transient focal cerebral ischemia in mice. However, Mincle was not widely expressed in brain. Its restricted pattern of expression may indicate a role for Mincle in regulating cerebral vasculature in response to injury, and thus help explain the apparent differences in phenotype of the Mincle KO mouse responding to brain MCAO or spinal cord neurotrauma.

Further clarification of Mincle's role in ischemic stroke requires major improvements to the available molecular toolkit for mouse. The use of the Mincle KO mouse as a negative staining control revealed serious deficits in most commonly used antibodies for tissue staining in mouse. Yet, it is on the strength of these antibodies that others have implicated Mincle in changes to innate immunity after ischemic stroke,^26^ subarachnoid haemorrhage^25^ and traumatic brain injury.^38^ We confirmed by multiple molecular methods (RNA and protein, in vitro and in vivo) that Mincle was not present in mouse or rat neurons. Microglia are radiation-resistant and thus retained in the recipient animal in bone marrow chimeras,^39^ and we had expected that these cells would be the most likely source of Mincle-directed inflammation; however, microglia isolated from sham-operated or tMCAO brains one day after the injury did not express the *Clec4e* mRNA, and the transcriptional profile of Mincle-deficient microglia did not deviate from that of isogenic controls, after sham surgery or ischemia. There were no differences in microglia-derived TNF production between WT and *Clec4e*^*−/−*^ animals after tMCAO, and microglia from WT mice were unresponsive to known Mincle-ligands.

The identity of the Mincle-positive cell associated with the brain vasculature is intriguing. The classical perivascular CD163^ + ^macrophage is thought to be a short-lived cell replenished from circulating CD14/CD16 monocytes, and although we have not formally demonstrated turnover of CD163^ + ^cells in our model, prior lineage studies support the likelihood that most perivascular macrophages are derived from bone marrow recipients in our chimera experiments.^40^ However, a subset of perivascular-resident macrophages (the perivascular macrophage-like melanocyte, PVM/M) are long-lived macrophages – less than 15% turnover of these cells was reported three months post bone marrow ablation and reconstitution.^41^ The PVM/M shares a similar vascular distribution to our pericyte-associated Mincle^ + ^cells, and are also intercalated with pericytes, extending processes to wrap around the blood vessel. PVM/M has been demonstrated as playing an important role in the quality of the blood–endothelial barrier in the inner ear.^42^ Further work is needed to confirm that the previously reported PVM/M expresses Mincle, that the Mincle^ + ^CD163^ + ^cells that we have reported here are equally long-lived, or that these cells have a role in the blood–brain barrier integrity in ischemic stroke; nevertheless, the longevity of this population is consistent with the phenotype observed from our own bone marrow chimera study, which would require the Mincle^ + ^CD163^ + ^perivascular cell to be resistant to the bone-marrow ablation and therefore maintaining the *Clec4e* genotype of the recipient mice.

The typical model for Mincle activation is as a necrotic cell receptor, and prior studies suggest that is active on CNS-resident cells.^25,26,38^ The inducible presence of Sap130 was also used as evidence for Mincle activity, but this is somewhat puzzling because Sap130 is a constitutively expressed nuclear protein, made available to immune receptors through cellular disruption via necrosis. Increased levels of Sap130 in whole tissue lysates are therefore most consistent with recruitment of additional cells to the lesion, perhaps due to influx of peripheral leukocytes.

Data presented here do not support the model of Mincle as a necrotic cell receptor. Mincle is not widely expressed on non-myeloid cells, and is not expressed on microglia in the days following tMCAO, so it is unlikely to be exerting its effects directly on neuronal survival or microglia activation. The lack of Mincle-dependent phenotypes after SCI, and gut and heart ischemia, challenges the widely held model of Mincle as a peripheral myeloid receptor directing inflammatory responses to areas of necrotic cell damage. The *Clec4e*^*−/−*^ mouse phenotype does not recapitulate that of other pattern recognition receptors with roles in necrotic cell recognition. For example, bone marrow chimera experiments using *Tlr2* and/or *Tlr4* showed that recipient KO mice were equally susceptible to tMCAO as WT mice. Functional benefit was only observed in donors receiving KO bone marrow, demonstrating that these receptors were most important in peripheral leukocyte activation.^43^ In contrast, our own chimera studies demonstrated that the lack of expression of Mincle on CNS-resident cells was protective regardless of the genotype of the donor marrow. Loss of Mincle on peripheral immune cells had a relatively small impact on the severity of MCAO. These data indicate that Mincle predominantly acts on an aspect specific to CNS architecture or function.

The significant reduction in brain infarct size in *Clec4e*^*−/−*^ animals suggests that blocking Mincle protects the cells in the penumbra region, that is, the brain region where blood perfusion is low but sufficient to maintain cell viability as a result of collateral blood vessel irrigation. Although we cannot fully exclude the possibility of anatomical differences in the development of the vasculature in *Clec4e*^*−/−*^ mice, the fact that blockade of Mincle signaling with a Syk inhibitor offered a similar functional benefit to genetic ablation of Mincle strongly argues against this, pointing towards functional differences in the response to the insult. Exhaustive analysis of SCI, where the blood–CNS barrier is disrupted by mechanical forces at the outset, revealed no Mincle-dependant effect. Certainly, many features of the CNS response to an ischemic injury do not apply to SCI, this includes the timing of recruitment of inflammatory infiltrate,^31^ as well as differences between the architecture and function of the blood–brain barrier compared to the blood–spinal cord barrier.^44^ In combination, these support our theory that Mincle acts specifically in instances where the integrity of the blood–CNS barrier is not directly compromised by the initiating event.

The direct physical association between Mincle^+^ perivascular macrophages and the adventitial plane of alpha-SMA^+^ pericytes is evidence that these cells may regulate brain microvasculature and/or breakdown of the blood–brain barrier following an ischemic event. The role of pericytes in the quality of blood flow following reperfusion has been recently highlighted,^45,46^ where activation of pericytes leads to vascular constriction and hyporeperfusion that exacerbates damage in the penumbra region of a stroke lesion. We speculate that Mincle expression by the pericyte-associated macrophage has an instructive role between brain injury and pericyte response.

An alternate and not mutually exclusive explanation would be that Mincle has a role in directing inflammatory cell recruitment across the blood–brain barrier, which may have contributed to the observed reduction in inflammatory infiltrate in the brains of *Clec4e*^*−/−*^ mice after stroke (Figure 2). This hypothesis is consistent with observations that neutrophils transit across endothelial barriers via pericyte processes,^47^ but it remains an open question as to whether the Mincle^ + ^macrophage associated with the pericyte contributes to the traffic of inflammatory monocytes, macrophages or neutrophils across the blood–brain barrier.

Although our results leave many unanswered questions about the mechanism by which blocking Mincle improves ischemic stroke outcomes, we demonstrate that it does have a dramatic influence over the outcome from brain ischemia and reperfusion injury, which mainly emerges from Mincle's presence in the brain itself. The apparent specificity of Mincle in exacerbating CNS injury and the likely availability of Mincle-expressing cells in the vasculature of injured brains provide the field with a new class of candidate in the search for modifiable targets of inflammation during stroke.

## Supplementary Material

Supplementary material
