# Supplementary material for: An atypical role for the myeloid receptor Mincle in central nervous system injury
Source: J Cereb Blood Flow Metab. 2016 Jan 1;37(6):2098–111. doi: 10.1177/0271678X16661201 (PMC5444551; doi:10.1177/0271678X16661201)
Supplement: Supplementary material [file 0271678x16661201.pdf]

Supplementary Figure 1. Mincle antibody staining on rat and mouse brain. (a) The rabbit anti-Mincle antibody (Bioss) did not reveal any clear signal on rat brain tissue. (b,c) Using alpha-SMA as a marker of pericytes and CD206 as a marker of perivascular macrophages, both rat anti-Mincle and mouse anti-Mincle antibodies reveal no specific staining in healthy brains from *Clec4e*$^{+/-}$ mice. (d) Background staining could be appreciated also in *Clec4e*$^{-/-}$ brains. Scale bar: 20 µm.

Supplementary Figure 2. The ARRIVE guidelines checklist for this study.

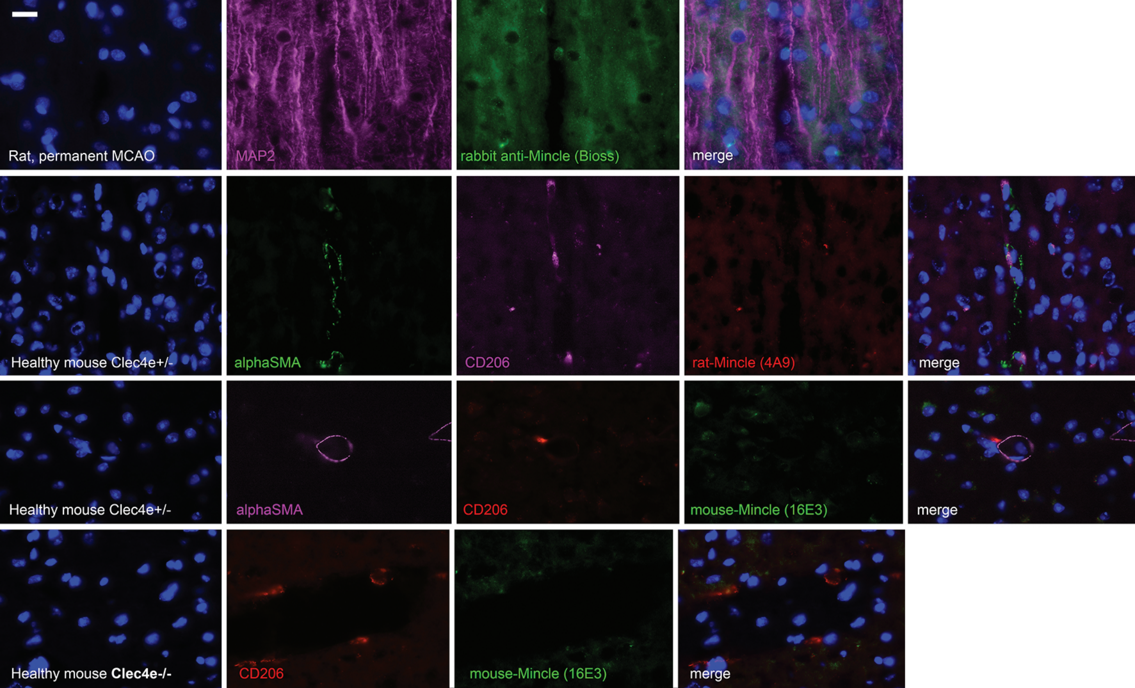
Rat, permanent MCAO
MAP2
rabbit anti-Mincle (Bioss)
merge
Healthy mouse Clec4e+/-
alphaSMA
CD206
rat-Mincle (4A9)
merge
Healthy mouse Clec4e+/-
alphaSMA
CD206
mouse-Mincle (16E3)
merge
Healthy mouse **Clec4e-/-**
CD206
mouse-Mincle (16E3)
merge

The ARRIVE Guidelines Checklist

Animal Research: Reporting In Vivo Experiments

Carol Kilkenny[1], William J Browne[2], Innes C Cuthill[3], Michael Emerson[4] and Douglas G Altman[5]

[1]The National Centre for the Replacement, Refinement and Reduction of Animals in Research, London, UK, [2]School of Veterinary Science, University of Bristol, Bristol, UK, [3]School of Biological Sciences, University of Bristol, Bristol, UK, [4]National Heart and Lung Institute, Imperial College London, UK, [5]Centre for Statistics in Medicine, University of Oxford, Oxford, UK.

	ITEM	RECOMMENDATION	Section/ Paragraph
Title	1	Provide as accurate and concise a description of the content of the article as possible.	Title
Abstract	2	Provide an accurate summary of the background, research objectives, including details of the species or strain of animal used, key methods, principal findings and conclusions of the study.	Abstract
**INTRODUCTION**			
Background	3	a. Include sufficient scientific background (including relevant references to previous work) to understand the motivation and context for the study, and explain the experimental approach and rationale.<br>b. Explain how and why the animal species and model being used can address the scientific objectives and, where appropriate, the study's relevance to human biology.	a)Paragraph 1-3<br>b)Paragraph 3-4
Objectives	4	Clearly describe the primary and any secondary objectives of the study, or specific hypotheses being tested.	Paragraph 4
**METHODS**			
Ethical statement	5	Indicate the nature of the ethical review permissions, relevant licences (e.g. Animal [Scientific Procedures] Act 1986), and national or institutional guidelines for the care and use of animals, that cover the research.	Paragraph 1
Study design	6	For each experiment, give brief details of the study design including:<br>a. The number of experimental and control groups.<br>b. Any steps taken to minimise the effects of subjective bias when allocating animals to treatment (e.g. randomisation procedure) and when assessing results (e.g. if done, describe who was blinded and when).<br>c. The experimental unit (e.g. a single animal, group or cage of animals).<br>A time-line diagram or flow chart can be useful to illustrate how complex study designs were carried out.	a,b) Paragraph 1, 3, 6, 7, 9, 10<br>c) Paragraph 15
Experimental procedures	7	For each experiment and each experimental group, including controls, provide precise details of all procedures carried out. For example:<br>a. How (e.g. drug formulation and dose, site and route of administration, anaesthesia and analgesia used [including monitoring], surgical procedure, method of euthanasia). Provide details of any specialist equipment used, including supplier(s).<br>b. When (e.g. time of day).<br>c. Where (e.g. home cage, laboratory, water maze).<br>d. Why (e.g. rationale for choice of specific anaesthetic, route of administration, drug dose used).	a,d) Paragraph 1, 3, 6, 7, 9, 10<br>b) Paragraph 1
Experimental animals	8	a. Provide details of the animals used, including species, strain, sex, developmental stage (e.g. mean or median age plus age range) and weight (e.g. mean or median weight plus weight range).<br>b. Provide further relevant information such as the source of animals, international strain nomenclature, genetic modification status (e.g. knock-out or transgenic), genotype, health/immune status, drug or test naïve, previous procedures, etc.	a)Paragraph 1, 3, 6, 7, 9, 10<br>b)Paragraph 1

Housing and husbandry	9	Provide details of:<br>a. Housing (type of facility e.g. specific pathogen free [SPF]; type of cage or housing; bedding material; number of cage companions; tank shape and material etc. for fish).<br>b. Husbandry conditions (e.g. breeding programme, light/dark cycle, temperature, quality of water etc for fish, type of food, access to food and water, environmental enrichment).<br>c. Welfare-related assessments and interventions that were carried out prior to, during, or after the experiment.	a,b)Paragraph 1<br>c)Paragraph 1, 3, 6, 7, 9, 10
Sample size	10	a. Specify the total number of animals used in each experiment, and the number of animals in each experimental group.<br>b. Explain how the number of animals was arrived at. Provide details of any sample size calculation used.<br>c. Indicate the number of independent replications of each experiment, if relevant.	a) Figures 1-5 (individual values plotted), Fig 1 legend<br>b)Paragraph 15
Allocating animals to experimental groups	11	a. Give full details of how animals were allocated to experimental groups, including randomisation or matching if done.<br>b. Describe the order in which the animals in the different experimental groups were treated and assessed.	a,b)Paragraph 1, 3, 6, 7, 9, 10
Experimental outcomes	12	Clearly define the primary and secondary experimental outcomes assessed (e.g. cell death, molecular markers, behavioural changes).	a,b) Paragraph 1, 3, 6, 7, 9, 10
Statistical methods	13	a. Provide details of the statistical methods used for each analysis.<br>b. Specify the unit of analysis for each dataset (e.g. single animal, group of animals, single neuron).<br>c. Describe any methods used to assess whether the data met the assumptions of the statistical approach.	Paragraph 15
RESULTS			
Baseline data	14	For each experimental group, report relevant characteristics and health status of animals (e.g. weight, microbiological status, and drug or test naïve) prior to treatment or testing. (This information can often be tabulated).	Methods paragraph 1
Numbers analysed	15	a. Report the number of animals in each group included in each analysis. Report absolute numbers (e.g. 10/20, not 50%[2]).<br>b. If any animals or data were not included in the analysis, explain why.	Methods paragraph 2
Outcomes and estimation	16	Report the results for each analysis carried out, with a measure of precision (e.g. standard error or confidence interval).	Figures 1-5
Adverse events	17	a. Give details of all important adverse events in each experimental group.<br>b. Describe any modifications to the experimental protocols made to reduce adverse events.	n.a.
DISCUSSION			
Interpretation/ scientific implications	18	a. Interpret the results, taking into account the study objectives and hypotheses, current theory and other relevant studies in the literature.<br>b. Comment on the study limitations including any potential sources of bias, any limitations of the animal model, and the imprecision associated with the results[2].<br>c. Describe any implications of your experimental methods or findings for the replacement, refinement or reduction (the 3Rs) of the use of animals in research.	Throughout discussion
Generalisability/ translation	19	Comment on whether, and how, the findings of this study are likely to translate to other species or systems, including any relevance to human biology.	Throughout discussion
Funding	20	List all funding sources (including grant number) and the role of the funder(s) in the study.	Detailed in “sources of support”

References:
1. Kilkenny C, Browne WJ, Cuthill IC, Emerson M, Altman DG (2010) Improving Bioscience Research Reporting: The ARRIVE Guidelines for Reporting Animal Research. *PLoS Biol* 8(6): e1000412. doi:10.1371/journal.pbio.1000412
2. Schulz KF, Altman DG, Moher D, the CONSORT Group (2010) CONSORT 2010 Statement: updated guidelines for reporting parallel group randomised trials. *BMJ* 340:c332.

	Figure	Experiment	Experimental units – used/planned: WT or Clec4e+/-, sham or control	Clec4e-/-, sham or control	Wild-type or vehicle	Clec4e-/-	Inhibitor pre	Inhibitor post	Outcome measure	Time points
***In vivo* experiments**	1b	Sham surgery or tMCAO 1 h	8/8	(-)	27/34	14/16	(-)	(-)	Infarct size	3 days
	1c	Sham surgery or tMCAO 1 h	8/8	(-)	27/34	14/16	(-)	(-)	Neurological deficit score	1, 2 and 3 days
	1d	tMCAO 1 h	(-)	(-)	8/14	8/12	(-)	(-)	Infarct size	7 days
	1e	tMCAO 1 h	(-)	(-)	8/14	8/12	(-)	(-)	Neurological deficit score	1, 2, 3, 4, 5, 6 and 7 days
	1f	Sham surgery or tMCAO 1 h	(-)	(-)	10/10	8/8	(-)	(-)	Blood flow %	0 and 1 day
	5d-f	Sham surgery or tMCAO 1 h	3/3	3/3	5/5	5/5	(-)	(-)	Microglia gene expression	1 day
	1g	Global I/R 30 min	4/4	(-)	6/6	6/6	(-)	(-)	TUNEL+ cells	3 days
	1i	Sham surgery or tMCAO 1 h	6/6	(-)	11/14	(-)	9/10	10/10	Infarct size	3 days
	1j	Sham surgery or tMCAO 1 h	(-)	(-)	11/14	(-)	9/10	10/10	Neurological deficit score	1, 2 and 3 days
	2a	tMCAO 1 h	(-)	(-)	5/5	5/5	(-)	(-)	Inflammatory infiltrate	1 day
	2a	tMCAO 1 h	(-)	(-)	7/7	7/7	(-)	(-)	Inflammatory infiltrate	3 days
	2b	tMCAO 1 h	(-)	(-)	3/3	3/3	(-)	(-)	TNF	1 day
	2c	tMCAO 1 h	(-)	(-)	4/4	(-)	(-)	3/3	Inflammatory infiltrate	1 day
	2c	tMCAO 1 h	(-)	(-)	3/3	(-)	(-)	3/3	Inflammatory infiltrate	3 days
	2d	tMCAO 1 h	(-)	(-)	4/4	(-)	(-)	3/3	TNF	1 day
			**WT or Clec4e+/-, sham or control**	**Clec4e-/-, sham or control**	**Wild-type or vehicle**	**Clec4e-/-**	**Clec4e-/- > WT**	**WT > Clec4e-/-**		
	3a	tMCAO 1 h	(-)	(-)	18/18	17/18	17/18	18/18	Infarct size	3 days
	3b	tMCAO 1 h	(-)	(-)	18/18	17/18	17/18	18/18	Neurological deficit score	1, 2 and 3 days
	4a,b	SCI	(-)	(-)	10/12	10/14	(-)	(-)	BMS score	0, 1, 4, 7, 14, 21, 28 and 35 days
	4d,e	SCI	(-)	(-)	9/12	10/14	(-)	(-)	Lesion volume and length	35 days
	4g	SCI	(-)	(-)	7/12	10/14	(-)	(-)	% myelin	35 days
	4h	Gut I/R 30 min	(-)	(-)	7/11	10/10	(-)	(-)	Gut pathology score	2 h
	4i	Gut I/R 30 min	(-)	(-)	9/11	8/10	(-)	(-)	MPO	2 h
	4l	Myocardial infarction	17/17	12/12	15/17	11/12	(-)	(-)	% EF	Before surgery and 30 days post surgery
	4m	Myocardial infarction	17/17	12/12	15/17	11/12	(-)	(-)	EVS	Before surgery and 30 days post surgery
	4n	Myocardial infarction	17/17	12/12	14/17	11/12	(-)	(-)	EVD	Before surgery and 30 days post surgery
	4o	Myocardial infarction	17/17	12/12	(-)	(-)	(-)	(-)	LV mass	Before surgery
***In vitro* experiments**	5b	Microglia stimulation	(-)	(-)	3/3	3/3	(-)	(-)	MIP2a mRNA	Unstimulated, 6 h OGD and 24 h TDM
	5c	Microglia stimulation	(-)	(-)	4/4	(-)	(-)	(-)	Clec4e mRNA	0, 1, 3, 5 and 9 hours OGD
	5g	Neuronal stimulation	(-)	(-)	4/4	(-)	(-)	(-)	Clec4e mRNA	0, 1, 3, 6 and 12 hours OGD
	5g	bEND.3 stimulation	(-)	(-)	2/2	(-)	(-)	(-)	Clec4e mRNA	0, 1, 3, 5 and 9 hours OGD
	5g	Astrocyte stimulation	(-)	(-)	2/2	(-)	(-)	(-)	Clec4e mRNA	0, 1, 3, 5 and 9 hours OGD

Materials and Methods

Animals and reagents. All experimental procedures followed the "Australian code of practice for the care and use of animals for scientific purposes", approved by The University of Queensland and Monash University Animal Ethics Committees (ethics license numbers SBMS/358/12/NHMRC/ARC, SBMS/085/09, MARP-2011-175 and SBMS/311/12/SPINALCURE), and further details, including ARRIVE guidelines for reporting animal research are available in the Supplementary methods. Leading up to experiments, all animals spent at least one week in conventional housing conditions (12h light/12h dark), with controlled temperature (22-26 ºC), humidity (40-60%), and *ad libitum* normal chow diet and water, autoclaved sawdust or corn cob for bedding and a maximum of 5 mice per cage. Male mice were individually housed. Animals were checked for health daily. Four different laboratories conducted the surgeries described herein. In all cases, except where indicated, a randomized experimental design consisted of pre-assigned groups of mice where the surgeon or operator was blind to genotype. All experiments took place in physical containment 2 (PC2) laboratories. All sections of this study adhere to the ARRIVE Guidelines for reporting animal research (Supplementary Figure 2).

PCR genotyping used primers: *ON14*, ATTGCCACTGACCCTCCACC; *MN469*: CCCCTGTCACTGTTTCTCTGCA; *MN473*: TGCAGCCCAAGCTGATCCTC.

Focal cerebral ischemia model. Male, 3 to 6 month-old mice were anesthetized for focal cerebral ischemia by transient middle cerebral artery occlusion (tMCAO). The first round of surgery (Figure 1 and Figure 2) did not use a randomized experimental design. For all other tMCAO, including bone marrow chimeras (Figure 3, n=70), and microglia profiling (Figure 5, n=16), operators were blinded to genotype or treatment group and randomization was based on predesigned lists using colour coded cages and reagents. Exclusion criteria were excessive

bleeding or death within 24 h after tMCAO. Table 1 summarizes the planned and actual animal numbers used in this study. On these grounds, 1 out of 10 Syk inhibitor pre-treated, 2 out of 16 *Clec4e*$^{-/-}$ and 7 out of 34 WT animals with tMCAO were excluded. Mice were anesthetized with 2% isoflurane in oxygen with spontaneous breathing and body temperature at 37°C. After a midline neck incision, the left external carotid and pterygopalatine arteries were isolated and ligated with 5-0 silk thread. The internal carotid artery was occluded with a clip at the peripheral site of the bifurcation to the pterygopalatine artery and the common carotid artery was then ligated with 5-0 silk thread. The external carotid artery was cut and a 6-0 nylon suture with a blunted tip (0.20 mm) was inserted. The clip at the internal carotid artery was then removed for advancement of the nylon suture into the middle cerebral artery to slightly more than 6 mm from the internal carotid-pterygopalatine artery bifurcation. After 1h, the nylon suture and ligatures were removed to initiate reperfusion for 24 h up to 7 days. In the sham group, these arteries were visualized but not disturbed. Animals were subjected to cerebral blood flow (CBF) measurements using a laser Doppler perfusion monitor (Moor Lab) to confirm MCAO. The Doppler laser tip was placed perpendicular to the surface of the right parietal skull (1 mm posterior and 5 mm lateral to the bregma) to monitor blood flow in the MCAO territory.

Quantification of cerebral infarction and neurological deficit assessment. At 3 or 7 days post-reperfusion, mice were euthanized, the brains removed into PBS (4ºC) for 15 min, and 2 mm coronal sections were obtained. These were stained with 2% 2,3,5-triphenyltetrazolium chloride (TTC, T8877 Sigma-Aldrich) at 37ºC for 15 min. The stained sections were photographed, and the images digitized. The infarct area of each section was defined as the pale area surrounded by red undamaged tissue. Measurements were done using NIH image 6.1 software. To correct for brain swelling, the infarct area was determined by subtracting the area of undamaged tissue in the left (ipsilateral) hemisphere from that of the whole contralateral

hemisphere. Infarct volume was calculated by integration of infarct areas for all slices of each brain, and then expressed as a % of the ipsilateral hemisphere. The functional consequences of tMCAO were evaluated using a 5-point neurological deficit score (0, no deficit; 1, failure to extend right paw; 2, circling to the right; 3, falling to the right; and 4, unable to walk spontaneously[29]).

Global ischemia model and TUNEL assay. Adult male, age-matched C57BL6/J WT (n=4) and *Clec4e*$^{-/-}$ mice (n=6) were used. Transient global cerebral ischemia was performed by the 2-vessel occlusion model[1]. The common carotid arteries were occluded for 30 minutes and the blood was allowed to reperfuse the tissue for 72 h before euthanasia. Mice were euthanized, brains were fixed and embedded in paraffin wax, and sections were pretreated with 20 µg/ml proteinase K (Roche, Switzerland) in 10 mM Tris, pH 7.4. The In Situ Cell Death Detection Kit, POD (11684817910, Roche, Switzerland) was used according to manufacturer's instructions, and tissue was counterstained with Gills haematoxylin. Sections treated with 100 U/ml recombinant DNase I were used as positive control.

Flow cytometry. Animals were euthanized and perfused with PBS prior to isolation of as described in [30]. Brains were dissected, cerebella removed, and the left ischemic (ipsilesional) hemispheres selected. Three hemispheres were pooled, digested for 30 min at 37°C (1 mg/ml collagenase, 0.1 mg/ml DNAse I in DMEM), and pressed through a 40 µm cell strainer, incubated with standard erythrocyte lysis buffer on ice, separated from myelin and debris, then incubated 30 min RT in buffer (0.5% bovine serum albumin, 0.02% sodium azide in PBS): CD45 (25-0451-82, eBioscience, 1:100), Ly6G (560603, BD Biosciences, 1:100), CD11b (557396, BD, 1:300), CD11c (17-0114-82, eBioscience, 1:100) and TNF (554419, BD, 1:100). Data were acquired with a LSR II FACS system (BD Biosciences) and analyzed with FlowJo (TreeStar). Doublets were excluded with FSC-A and FSC-H linearity.

Spinal cord injury and assessment of locomotor recovery. Adult, age- and weight-matched female C57BL6/J WT (n=12) and *Clec4e*$^{-/-}$ mice (n=14) were used for these experiments. Order of surgery was randomized based on predesigned lists, with the experimenter conducting the surgery remaining blinded to genotype throughout all aspects of surgery. In brief, mice were anesthetized via intraperitoneal injection with Xylazine (10 mg/kg, Ilium) and Zolazepam (50 mg/kg, Virbac) and subjected to a severe contusive SCI. The ninth thoracic (T9) vertebra was identified as described previously[31], followed by a dorsal laminectomy as described previously[32,33]. A force-controlled 70 kilodyne (kd) impact was applied at spinal level T11, using the Infinite Horizon impactor device (Precision Systems and Instrumentation). Paravertebral muscles were sutured post-impact. SCI mice were then randomly re-assigned to cages labelled A, B, C, etc., with 2-3 mice being housed per box. Post-operative care involved sub-cutaneous administration of a single dose of buprenorphine (0.05 mg/kg) in Hartmann's Sodium Lactate solution for analgesia. A prophylactic dose of Gentamicin, (1.0 mg/kg, Ilium) was administered daily for 5 days post-injury. Recovery of hind-limb function was assessed using the 10-point Basso Mouse Scale (BMS), a system designed specifically for the assessment of murine locomotor recovery following SCI[34]. Multiple aspects of locomotion, including ankle movement, stepping, co-ordination, paw placement, trunk stability and tail position, were assessed using this scale. Experimental animals were randomly picked up from their cages and assessed by two investigators blinded to the genotype at 1, 4, 7 days post-SCI and then weekly thereafter up until the study endpoint (35 days post-injury). Animals deviating > ±5 kdyne from the mean force, or spinal cord tissue displacement ±100 μm from the experimental mean were excluded. 2 WT and 4 *Clec4e*$^{-/-}$ mice were excluded from the study under these criteria. For the remaining n=10 mice per genotype, the actual applied force and displacement for WT and *Clec4e*$^{-/-}$ animals was 75.40±0.91 *vs*. 74.10±0.99 kdyne ($p>0.34$), and 537.7±15.22 *vs*. 535.9±18.11 μm, respectively ($p>0.94$).

Spinal cord tissue sectioning and immunofluorescence. All mice were euthanized at 35 days post-injury. In brief, mice were deeply anesthetized using sodium pentobarbital and transcardially perfused with 15ml of saline (0.9% NaCl containing 2IU/ml Heparin (Pfizer) and 2% $NaNO_2$), followed by 30ml of phosphate-buffered Zamboni's fixative (2% Picric acid, 2% Formaldehyde, pH 7.2-7.4). Vertebral columns were excised and post-fixed overnight at 4°C. The spinal cord was dissected and placed in sequential overnight incubations of 10% and 30% sucrose in PBS, followed by embedding in Tissue-Tek Optimal Cooling Temperature (Sakura Finetek), and snap-freezing on dryice-isopentane. Transverse 20 μm thick sections of spinal cord were cut using a Leica Cryostat CM3050-S and collected in 1:5 series on Superfrost Plus slides. Sections were incubated 1 h RT with IHC blocking buffer (2% Bovine Serum Albumin and 0.2% Triton X-100) in a humidified chamber, then overnight at 4°C with primary antibodies: 1:1600 chicken anti-mouse GFAP (Abcam; #ab4674) and 1:200 rabbit anti-mouse fibronectin (Sigma-Aldrich; #F3648), or 1:1000 rabbit anti-mouse GFAP (Dako; #Z0334) in the absence of fibronectin staining. After washing, slides were incubated for 1 h at RT with the following secondary antibodies as required: 1:400 goat anti-chicken 555 (Abcam; #ab150170) and 1:400 goat anti-rabbit 488 (Thermo Fisher Scientific; #A-11034) and 1:150 FluoroMyelin Red (Thermo Fisher Scientific; #F34652). Hoechst 33342 nuclear dye was used for counterstaining. Images were captured on a single plane using a Zeiss Axio Imager and Zen Blue 2012 Software (Zeiss), and analysed with ImageJ software. Section areas were determined by outlining the section boundary on the $GFAP^+$ channel (excluding the leptomeninges). Proportional area measurements were calculated by thresholding the FluoroMyelin Red stained area in ImageJ and dividing it by the total section area. Lesion volumes and/or length were calculated by multiplying $fibronectin^+$ areas by the section thickness and 1:5 series count.

Intestinal ischemia and reperfusion, histological analysis and myeloperoxidase (MPO) quantification. Mice (WT, n = 11; *Clec4e*$^{-/-}$, n = 10) were anesthetized with 2% isoflurane in

oxygen through a facemask, with spontaneous breathing and body temperature at 37°C. The surgeries were not randomized, but tissues were collected into coded tubes and the analysis performed by an operator blind to genotype. A midline incision was made through the skin and then along the linea alba separating the rectus abdominis muscle. The exposed intestines were displaced and a ligature was tied with silk suture material around the superior mesenteric artery except in animals undergoing sham surgery. After 30 min of ischemia the ligature was removed, and after 2 h of reperfusion the mice were euthanized. For histological analysis, three portions of small intestine were stored in 4% paraformaldehyde for 24 h. Tissues were embedded in paraffin wax, sectioned transversely and stained with haematoxylin/eosin. The average of villi damage was determined after grading each of 100 villi per mouse on a 0–6 scale as previously described[35]. For MPO activity, three portions of small intestine were homogenized in 50 mM potassium phosphate, centrifuged and pellets resuspended in 0.25 mM hexadecyltrimethylammonium bromide (H5882, Sigma-Aldrich) for MPO solubilisation. After homogenisation and centrifugation, supernatants were assayed with 1.21 mg/ml o-dianisidine dihydrochloride (D3252, Sigma-Aldrich) and 2.17% hydrogen peroxide, and absorbance read at 460 nm.

Myocardial infarction and echocardiography analysis. All animals in the same cage (siblings) were experimented on in a blinded fashion (for both echocardiography and surgery). Genotypes were checked after the experiment was finalized. To induce myocardial infarction, the left coronary artery (LDCA) of 10 week-old mice was ligated. For this procedure, animals were anaesthetized using 2% isoflurane and subjected to artificial ventilation through endotracheal cannulation. An incision was made through the muscle of the 4th and 5th intercostal space, and an 8-0 polyethylene suture passed under and tied around the LDCA 1 mm below the tip of the left auricle. Buprenorphine analgesic solution was administered subcutaneously (0.05 μg/g) twice a day for 3 days following surgery. For non-invasive

echocardiography, control (heterozygous or WT) and mutant (homozygous) adult mice, in homeostasis or 1 month after surgical intervention, were anaesthetized and kept sedated under 1.5% isoflurane. Imaging was performed in spontaneously breathing animals in prone position using Vevo 2100 Imaging System (FUJIFILM VisualSonics) equipped with 18 to 38 MHz linear array transducer. Standard parasternal long- and short-axis views were obtained to assess left ventricular chamber function. Breathing was monitored to avoid measurement distortions during breathing cycle. Calculations of cardiac function were done using Vevo2100 Cardiac Measurements Package. Mice were euthanized one month after surgery, their hearts dissected for histology.

Cell culture, and oxygen and glucose deprivation. Neuronal cultures were established from littermate 16 day-old WT, *Clec4e*$^{+/-}$ or *Clec4e*$^{-/-}$ mouse embryos. Genotypes were checked after the experiment was finalized. Cells were maintained at 37 °C in Neurobasal medium containing Glutamax and B-27 supplements, and 0.001% gentamycin sulfate (Life Technologies). Cells were ascertained by immunofluorescence to be 95% neurons and 5% astrocytes, with the occasional microglia. Glial cultures were established from postnatal day 1 WT, Clec4e$^{+/-}$ or Clec4e$^{-/-}$ mice and seeded in DMEM/F12 medium containing Glutamax and gentamycin 10 mg/L (all Life Technologies), and 10% fetal bovine serum (FBS). Microglia were separated from astrocytes with the use of CD11b (Microglia) MicroBeads (Miltenyi Biotec). The murine brain endothelial cell line bEnd.3 (ATCC CRL-2299) was grown in DMEM supplemented with Glutamax (Life Technologies). For oxygen and glucose deprivation (OGD), cultures were incubated with glucose-free Locke's buffer (in mmol/L: 154 NaCl, 5.6 KCl, 2.3 $CaCl_2$, 1 $MgCl_2$, 3.6 $NaHCO_3$, 5 HEPES, pH 7.2, supplemented with gentamycin 10 mg/L), placed in an incubator where the oxygen was displaced with nitrogen to a level of 0.2%, and incubated for 3 hours. Incubation with trehalose dimycolate (Sigma Aldrich) was conducted for 24 hours.

Immunofluorescence. Primary microglia or RAW264.7 cells (ATCC TIB-71) were grown on 12 mm coverslips, fixed in 4% paraformaldehyde. 6 μm microtome sections from Wistar Kyoto or spontaneously hypertensive stroke-prone (SHRSP) rats subjected to permanent MCAO for 24 h using diathermy with modification were obtained from a previous study[36]. Primary antibodies: rat anti-Mincle (clone 1B6, clone 4A9, D292-3M2, MBL), mouse anti-Mincle (clone 16E3, ab100846, Abcam); mouse alpha-SMA, (clone 1A4, ab7817, Abcam); rabbit GFAP (polyclonal, ab4674, Abcam), mouse anti-CD163 (clone ED2, Santa Cruz Biotechnology, sc-59865), goat anti-Iba1(polyclonal, ab5076, Abcam). Secondary antibodies were conjugated with Alexa Fluor 488, 568 and 647 (Life Technologies). Hoechst 33342 nuclear dye was used for counterstaining. Images were acquired using an Olympus BX61 microscope (Japan).

Microglia isolation and microarray. WT and *Clec4e*$^{-/-}$ mice were coded and randomized for tMCAO surgery and FACs profiling as described above. At 24hr, mice were perfused with PBS, their brains dissected, and 2 ipsilesional hemispheres (with cerebellum and brainstem removed) pooled for microglia isolation. For sham-operated animals, the whole forebrain was used and brains were not pooled. Tissue was minced with a razor blade, triturated by pipetting up and down gently 20 times, and pressed through a 40 μm cell strainer; all steps were carried out on ice. After myelin separation by Percoll gradient centrifugation, 80,000 CD45^{intermediate}, CD11b^{+} microglial cells were sorted from each sample. Doublets were excluded with FSC-A and FSC-H linearity, and dead cells excluded using Zombie Violet™ Fixable Viability Kit (423113, BioLegend). RNA was isolated with RNeasy Micro Kit (74004, Qiagen), and yield and quality measured with the RNA 6000 Pico Kit (Agilent Technologies, 5067-1513) for Agilent Bioanaliser. Yield ranged between 1.4 and 11 ng, RNA integrity number ranged between 8.1 and 10. Samples were amplified with the GeneChip WT Pico Kit (902623, Affymetrix) processed with the Mouse 2.0ST Gene Array WT pico assay (902463, Affymetrix)

by the Ramaciotti Centre for Genomics, University of New South Wales. The expression data (RMA background corrected, quantile normalized) is hosted by www.stemformatics.org (dataset S4M-6731)[37] and is available from GEO (Accession GSE77986). The expression threshold was calculated as the median expression of all antigenomic probesets on the microarray ($\log_2$ 3.38). Probes that failed to be expressed above detection threshold in the majority of biological replicates in at least one comparison group were removed from the analysis. The R/Bioconductor *limma*[38] package was used to find differentially expressed genes (DEG) at FDR P< 0.01.

Antibody	Application	Supplier	Dilution
CD45	Flow cytometry from fixed and perfused tMCAO mouse brains	25-0451-82, eBioscience	1:100
Ly6G		560603, BD Biosciences	1:100
CD11b		557396, BD	1:300
CD11c		17-0114-82, eBioscience	1:100
TNF		554419, BD	1:100
GFAP	Spinal cord tissue sectioning and immunofluorescence	ab4674, Abcam	1:1600
fibronectin	Spinal cord tissue sectioning and immunofluorescence	F3648, Sigma-Aldrich	1:200
GFAP	Spinal cord tissue sectioning and immunofluorescence in the absence of fibronectin staining	Z0334, Dako	1:1000
Rat anti-Mincle	Characterisation of Mincle cell in brain	clone 1B6, clone 4A9, D292-3M2, MBL	IF
mouse anti-Mincle		clone 16E3, ab100846, Abcam	IF

alpha-SMA		clone 1A4, ab7817, Abcam	IF
CD163		clone ED2, Santa Cruz Biotechnology, sc-59865	IF
Iba1		ab5076, Abcam	IF

Target	Forward primer	Reverse primer
Mouse Clec4e mRNA expression	TGCTACAGTGAGGCATCAGG	GGTTTTGTGCGAAAAGGAA
Mouse Mip2a mRNA expression	GAGACGGGTATCCCTTCGAC	TTCAGGGTCAAGGCAAACTT
